# Induction of activating transcription factor 3 (ATF3) in the cerebral cortex of a mouse model of blast-induced traumatic brain injury

**DOI:** 10.1007/s13577-026-01377-1

**Published:** 2026-04-07

**Authors:** Arumu Endo, Yasushi Satoh, Minori Koga, Wataru Nagata, Ken Yokochi, Fumiho Asai, Akihiro Ebihara, Tomohiro Tsuru, Yoshiyuki Araki, Masashi Kashitani, Hiroyuki Toda, Nozomi Ito, Machiko Kawasaki, Toshiaki Ishizuka, Kojiro Wada

**Affiliations:** 1 Department of Neurosurgery, The National Defense Medical College, Saitama, Japan; 2 Department of Biochemistry, The National Defense Medical College, 3-2 Namiki, Tokorozawa, Saitama 359-8513 Japan; 3 Department of Psychiatry, The National Defense Medical College, Saitama, Japan; 4 Department of Pharmacology, The National Defense Medical College, Saitama, Japan; 5 Military Medicine Research Unit, Test and Evaluation Command, Japan Ground Self-Defense Force, Setagaya, Tokyo Japan; 6 Department of Defense Medicine, The National Defense Medical College, Saitama, Japan; 7 Department of Aerospace Engineering, National Defense Academy, Kanagawa, Japan

**Keywords:** Traumatic brain injury, Blast, Activating transcription factor 3 (ATF3), Glia, Neuroinflammation

## Abstract

Traumatic brain injury (TBI) is a leading cause of long-term disabilities and mortality worldwide. Blast-induced TBI (bTBI) is the injury most commonly sustained by military personnel, but the pathomechanisms are largely unknown. Recently, accumulating evidence has suggested that neuroinflammation, characterized by the activation of microglia and astrocytes and elevated production of inflammatory mediators such as interleukins, cytokines, and chemotactic cytokines (chemokines), is a key pathological feature in bTBI. Therefore, controlling excessive neuroinflammation is critical to improve long-term neurological outcomes after bTBI, so understanding the mechanism of the neuroinflammation in bTBI is of significant interest. Activating transcription factor 3 (ATF3) is one of the most important transcription factors that regulate local and systemic inflammation in multiple pathophysiological processes such as cardiovascular disease, dementia, and ischemia/reperfusion-induced damage. Recently, ATF3 has attracted much attention for involvement in the neuroinflammatory response, by regulating the production of neuroinflammatory mediators, in a weight-drop–based TBI model. In this model, the upregulation of *Atf3* messenger ribonucleic acid (mRNA) levels was rapidly induced with the strongest increase at 1–2 h and decline by 4 h post-injury. However, there is little information about ATF3 in bTBI, and thus, the present study examined the expression of ATF3 in a mouse bTBI model. We here show the significant upregulation of *Atf3* mRNA was not observed in the cerebral cortex at 2 h post-exposure. However, the upregulation was observed at 5 days post-injury. Our results suggest robust differences in time course of neuroinflammation between other non-blast and blast TBIs.

**Supplementary Information:**

The online version contains supplementary material available at 10.1007/s13577-026-01377-1.

## Introduction

Traumatic brain injury (TBI) can be characterized into various types based on the causative forces. Even lower levels of exposure to blast can cause blast-induced TBIs (bTBIs), resulting in chronic neurological deficits including psychiatric impairments [1–4]. The cellular mechanisms underlying bTBI are less well understood, but may involve a broad spectrum of cellular pathways similar to other types of TBI. TBI induces strong activation of inflammatory responses, and the resultant brain immune dysregulation initiates a cascade of inflammatory signaling events, ultimately resulting in neuroinflammation [5]. Neuroinflammation is a critical process known to occur in various types of TBI, and such neuroinflammation can persist for years, and further trigger secondary neurodegeneration due to the excessive neuroimmune reaction affecting the long-term neurological outcome of affected patients. Therefore, control of disproportionate neuroinflammation is emerging as a candidate therapeutic strategy for improving neurological function after TBI. However, recent evidence suggests that bTBI may involve some pathological processes distinct from other non-blast TBIs [6]. Therefore, the differences in the mechanisms of neuroinflammation caused by blast versus other non-blast TBIs must be understood to improve long-term neurological outcomes.

Activating transcription factor 3 (ATF3) belongs to the cyclic adenosine monophosphate response element-binding protein (CREB) family [7]. ATF3 regulates the transcription of numerous proinflammatory cytokines containing ATF/CREB binding sites [8]. Recently, ATF3 has attracted much attention for its key involvement in neuroinflammation associated with several TBIs [9, 10]. Analysis of protein–protein interaction revealed that ATF3 interacts with numerous important stress-relevant proteins including transcriptional complexes involved in inflammatory responses, indicating that ATF3 is a hub of the cellular adaptive-response networks modulating metabolism and immunity [11]. Moreover, dysregulation of ATF3 for inflammatory responses exacerbates neuroinflammation resulting in poorer outcomes of TBI [9, 12]. Interestingly, mice deficient for ATF3 exhibit enhanced TBI-induced paresis and hematoma formation, suggesting that ATF3 negatively regulates the transcription of numerous proinflammatory cytokines and limits these injury outcomes in wild-type mice [9]. In addition, ATF3 may interact with a complex metabolic-immune response network in various cells, such as astrocytes and microglia, further suggesting that ATF3 is an important transcription factor in TBI [8, 13]. Therefore, transcription and metabolic regulation by ATF3 are recognized as powerful factors controlling neuroinflammation in TBI [8, 14, 15].

The course of common types of TBI-associated brain damage can be classified into two main phases [16]. The acute phase, the initial primary damage phase which occurs immediately after insult, involves vascular pathology, brain swelling, immune cell activation, release of inflammatory mediators, diffuse axonal injury, necrotic cell death, and breakdown of the blood–brain barrier [16–18]. However, these mechanisms cannot explain the clinical deterioration associated with TBI, as a large percentage of deaths caused by TBI do not occur in this acute phase but rather in a subsequent extended subacute phase that follows the acute phase over days to weeks after the event and may persist over much longer times. The subacute phase is caused by a broad spectrum of cellular and molecular responses including glutamate excitotoxicity, oxidative stress, and persistent neuroinflammation [19–22], which can all occur time-dependently. In particular, neuroinflammation can be present in both the acute and subacute phases of TBI [23].

Neuroinflammation appears to be responsible for beneficial effects which repair damaged tissue as well as for detrimental effects. Beneficial effects can occur if the inflammation is controlled in a regulated manner and for a defined period of time [16]. However, sustained or excessive neuroinflammation can become a major cause of numerous neuropathologies and may lead to progressive neurodegenerative processes [23, 24]. Therefore, the time-dependence of neuroinflammation is highly important. Poorly controlled time-dependence of neuroinflammation will dramatically worsen the primary injury damage. This importance of the time-dependence of neuroinflammation has led to investigation of the regulation of the time-dependent activation of ATF3.

ATF3 exhibits rapid but transient activation in the acute phase in the impact area of the brain of the closed-head weight-drop–TBI model [9]. In this model, the *Atf3* messenger ribonucleic acid (mRNA) levels were rapidly upregulated with the strongest increase at 1–2 h and decline by 4 h post-injury [9]. However, the regulation of ATF3 in bTBI is little documented. We previously showed that several bTBI-inflicted processes observed in humans can be reproduced in rodent bTBI models using our blast tube method [2, 25, 26]. The present study examined whether ATF3 is expressed after blast exposure in the mouse bTBI model using the blast tube method.

## Materials and methods

### Animals

All experiments were conducted according to the institutional ethical guidelines for animal experiments of the National Defense Medical College (Tokorozawa, Saitama, Japan) and were approved by the Committee for Animal Research at the National Defense Medical College (Approval No. 21046).

Age-matched male littermate C57BL/6 mice at 8 weeks of age were used in this study. The mice were housed under standard laboratory conditions with a 12-h light/dark cycle and a room temperature maintained at 23 ± 1˚C. The mice had ad libitum access to water and food.

### Shock tube setting

**Fig. 1:**
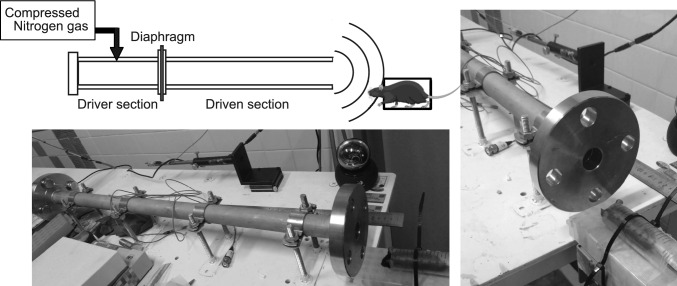
Experimental setting for the mouse model of blast-induced TBI. The shock tube consists of the driver and driven sections separated by a diaphragm. A mechanically driven needle pierces the diaphragm resulting in instant release of compressed nitrogen gas in the driver section causing a shock wave to propagate to the driven section and the anesthetized mouse placed on the holder

We used a compressed-gas driven shock tube as previously described [2]. The shock tube consists of the driver and driven sections, which are separated by a polyester diaphragm (Fig. 1). The driver section is filled with compressed nitrogen through the gas inlet valve, which is closed once the driver section is filled. Nitrogen gas flow is used to remove any water condensation in the process. Triggering of the device causes a needle to rupture the polyester diaphragm, which allows the driver gases to expand rapidly forming a shock wave traveling along the driven section. The pressure in the shock wave was measured at a resonant frequency of 0.5 MHz via a piezoelectric sensor (Model 113B26; PCB Piezotronics, Depew, NY). The analog output from the piezoelectric sensor was analyzed and recorded with an oscilloscope (DSO7104A; Agilent Technologies, Santa Clara, CA) using a 20 M Sa/s sample rate.

Animals were anesthetized before blast exposure by intraperitoneal injection of mixed anesthetic agents containing 0.3 mg/kg dexmedetomidine hydrochloride (Domitol; Nippon Zenyaku Kogyo, Kouriyama, Fukushima, Japan), 4.0 mg/kg midazolam (Midazolam; Sandoz, Tokyo, Japan), and 5.0 mg/kg butorphanol tartrate (Vetorphale; Meiji Seika Pharma, Tokyo, Japan). The animal was placed on a mouse placement holder at a distance of 5 cm from the exit end of the blast tube, with the head directed toward the blast tube and the long axis of the body parallel to the blast tube (Fig. 1). Control mice were sham-operated in the same room.

### Total RNA extraction and complementary deoxyribonucleic acid (cDNA) synthesis

Total RNA extraction and cDNA synthesis were carried out as previously described [26]. Briefly, after mice were anesthetized using the anesthetic mixture described above for blast exposure, cardio-perfusion with 0.1 M phosphate-buffered saline (PBS) was performed to remove the blood. Next, the brains were extracted and immersed in RNAlater solution (Thermo Fisher Scientific, Waltham, MA). Total RNA (1 μg) was isolated using the RNeasy Lipid Tissue Mini Kit (Qiagen, Hilden, Germany) to synthesize cDNA with ReverTra Ace® qPCR RT Master Mix (Toyobo, Osaka, Japan). The cDNA was diluted 1:10 with ultra-pure water, then the same volume of 5 M betaine (Sigma-Aldrich, St. Louis, MO) was added.

### Real-time polymerase chain reaction (PCR)

Quantitative real-time PCR was carried out as previously described [26], using the Thunderbird SYBR qPCR mix (Toyobo) in accordance with the manufacturer's instructions on the QuantStudio 5 Real-Time PCR System (Thermo Fisher). The amplification parameters were as follows: initial incubation of 95 °C for 1 min, followed by 50 cycles of denaturing at 95 °C for 15 s, and annealing and extension at 60 °C for 1 min. For each cycle, the fluorescent emission of SYBR green from each sample was recorded and calculated to determine the threshold cycle (C_t_) numbers. The primer sets (Table 1) for the genes were chosen from Harvard Medical School’s PrimerBank (https://pga.mgh.harvard.edu/primerbank/). The relative fold changes (2^−ΔΔCt^) in each experimental group compared to the control group were calculated in accordance with the 2^−ΔΔCt^ method [27]. β-Actin expression was unaltered after injury (Fig. S1), so the levels of mRNA expression were normalized to those of the corresponding β-actin as a reference gene. We examined 8 mice per condition (total 32 mice) but one analysis was excluded since the melting curve analysis showed a dissociation curve with two peaks, one of which corresponded to primer dimers.

**Table 1:** primers used for real-time PCR

Gene	Forward primer (5'–3')	Reverse primer (5'–3')
Atf3	GAGGATTTTGCTAACCTGACACC	TTGACGGTAACTGACTCCAGC
Gfap	CGGAGACGCATCACCTCTG	AGGGAGTGGAGGAGTCATTCG
Iba1	ATCAACAAGCAATTCCTCGATGA	CAGCATTCGCTTCAAGGACATA
β-actin	GGCTGTATTCCCCTCCATCG	CCAGTTGGTAACAATGCCATGT

### Immunohistochemistry

Immunohistochemical studies were performed as previously described [28]. Briefly, following anesthesia, mice were transcardially perfused with ice-cold PBS followed by 4% paraformaldehyde (PFA) in PBS, and the brains were extracted and eembedded in paraffin. Paraffin Sects. (5 µm-thick) were deparaffinized with xylene and rehydrated with ethanol, then immersed in unmasking solution (H-3300; Vector Laboratories, Newark, CA) for antigenic retrieval and heated in an autoclave (121°C) for 5 min. After incubated with blocking reagent (Nacalai Tesque, Kyoto, Japan), sections were incubated with primary antibodies at 4 °C overnight. The primary antibodies were rabbit polyclonal anti-glial fibrillary acidic protein (GFAP) (BS-0199R; Thermo Fisher), goat polyclonal anti-Iba1 (011–27991, Wako, Osaka, Japan), mouse monoclonal anti-neuronal nuclei (NeuN) (MAB377; Millipore, Burlington, MA), or rabbit monoclonal anti-ATF3 (ab305293; Abcam, Cambridge, UK) antibodies. The secondary antibodies used were AlexaFluor 488-conjugated goat anti-mouse immunoglobulin G (IgG) (Invitrogen, Carlsbad, CA), AlexaFluor 488-conjugated donkey anti-goat IgG (Invitrogen), or AlexaFluor 546-conjugated anti-rabbit IgG (Invitrogen) secondary antibodies which were incubated for 60 min at room temperature. Sections were examined with a confocal fluorescence microscopy using C2 Confocal Laser Microscope (Nikon, Tokyo, Japan). The Atf3-positive cells in a square of 214 × 214 μm in the parietal cortex were counted, and averages of four squares per each mouse were calculated. Samples from at least six mice per condition were examined in each experiment.

### Statistical analysis

Statistical analyses were performed using GraphPad Prism 9.5.1 (GraphPad Software, La Jolla, CA). To compare the means of four groups, conformity to the normal distribution was examined using the Shapiro–Wilk test, and equality of variances was examined using the F test. Those that were found to violate normality or equal variance were log-transformed and re-tested. Two-way analysis of variance (ANOVA) with Holm-Šidák multiple-comparison test was used if the data met the assumption of normality and equal variance. If the data do not meet the assumption, Games-Howell multiple-comparison test was performed. The results are presented as the mean ± standard error of the mean. The significance level was set at less than 0.05, indicating that *p* values below this threshold were considered statistically significant.

## Results

### Pressure–time profiles of the blast waves

**Fig. 2:**
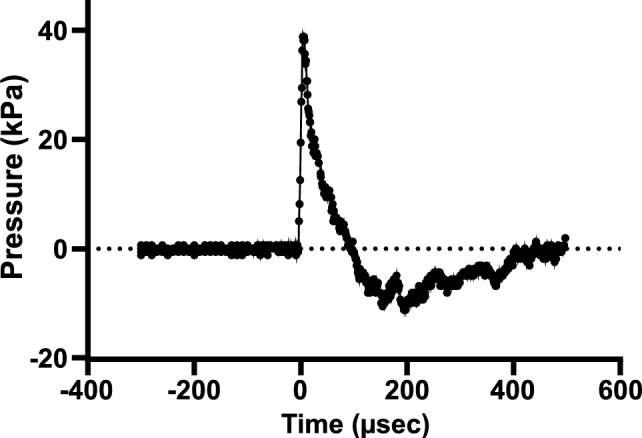
Pressure–time profiles of the blast waves. Typical blast waveform measured at the mouse head with the pressure in the driver section of 2.5 MPa. The blast wave consisted of a steep shock wavefront of positive overpressure, followed by positive and negative pressure phases

Figure 2 shows the pressure–time profiles of the blast waves at the animal point (mouse head) in the present study (pressures of driver section was 2.5 MPa). The generated pressure–time profile was reproducible. The waveform consisted of a steep front corresponding to the shock wave with a peak overpressure of 40 kPa, followed by decay of overpressure and negative pressure phases (Fig. 2). bTBI varies in severity from mild to severe. Blast overpressures less than approximately 150 kPa are classified the range as inducing "mild" bTBI [29–34], so this study adopted conditions in the range of "mild” bTBI.

### ATF3 expression is upregulated at the subacute phase after blast exposure

**Fig. 3:**
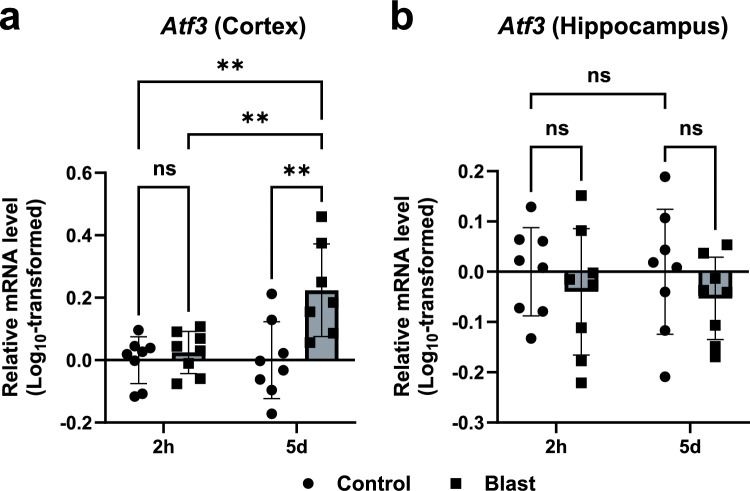
*Atf3* mRNA levels slowly induced after blast exposure. **a** At 2 h after blast exposure, *Atf3* mRNA levels were not obviously modulated in the cortex compared to the controls. In contrast, at 5 days after blast exposure, *Atf3* mRNA levels were upregulated in the cortex compared to the controls (two-way ANOVA with Holm-Šidák multiple-comparison test, control (2h): n = 8, bTBI (2h): n = 8, control (5d): n = 8, bTBI (5d): n = 7). **b** *Atf3* mRNA levels in the hippocampus were indistinguishable between the groups at 2 h or 5 days after blast exposure (two-way ANOVA with Holm-Šidák multiple-comparison test, n = 8 for each). Data are presented as mean ± standard error of the mean

Firstly, we analyzed the mRNA levels of *Atf3* 2 h post-injury since the strongest increase was observed at 1–2 h, but decline by 4 h post-injury in a previous study with non-blast TBI model [9]. Total RNA was obtained from the cerebral cortex and hippocampus, and the levels of *Atf3* mRNA were analyzed using real-time PCR. At acute phase (2 h post-exposure), the mRNA levels of *Atf3* were indistinguishable between bTBI and control groups in the cerebral cortex (Fig. 3a). In contrast to the result of acute phase, a significant increase was observed in the *Atf3* mRNA levels in the cerebral cortex of mice in the subacute phase (5 days post-exposure) compared to those of controls (Fig. 3a). These results were confirmed by a two-way ANOVA with Holm-Šidák multiple-comparison test (control (2h): n = 8, bTBI (2h): n = 8, control (5d): n = 8, bTBI (5d): n = 7), indicating the significant main effect of blast exposure (control vs. bTBI,* F* (1, 27) = 10.44, *p* = 0.0032), significant main effect of time (2h vs. 5 d,* F* (1, 27) = 6.685, *p* = 0.0154), and significant interaction effect (*F* (1, 27) = 6.683, *p* = 0.0155). In the hippocampus, there were no main effects (for either blast exposure or time) and no significant interaction effect (Fig. 3b; all *p* > 0.05, two-way ANOVA, n = 8 for each) in the mRNA levels of *Atf3*. These results demonstrated that mRNA levels of *Atf3* in the cortex were upregulated during a subacute phase, but not in acute phase in a mouse bTBI model.

### ATF3 was expressed mainly in the neurons after blast exposure

**Fig. 4:**
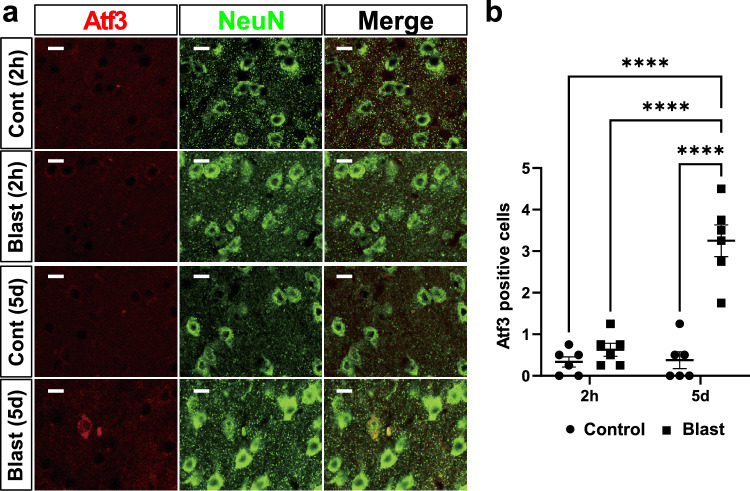
ATF3 immunoreactivity in the cerebral parietal cortex. **a** At 2 h after blast exposure, ATF3 immunoreactivity was indistinguishable between the control and bTBI groups in the cerebral parietal cortex. At 5 days after blast exposure, ATF3-positive cells increased in number and were colocalized with neuronal nuclei (NeuN). NeuN: neuronal nuclei. Scale bars: 10 μm. **b** Average numbers of ATF3-positive cells of panels. n = 6 for each group. n = 6 for each group. ****p < 0.0001 (two-way ANOVA with Holm-Šidák multiple-comparison test). Data are presented as mean ± standard error of the mean

**Fig. 5:**
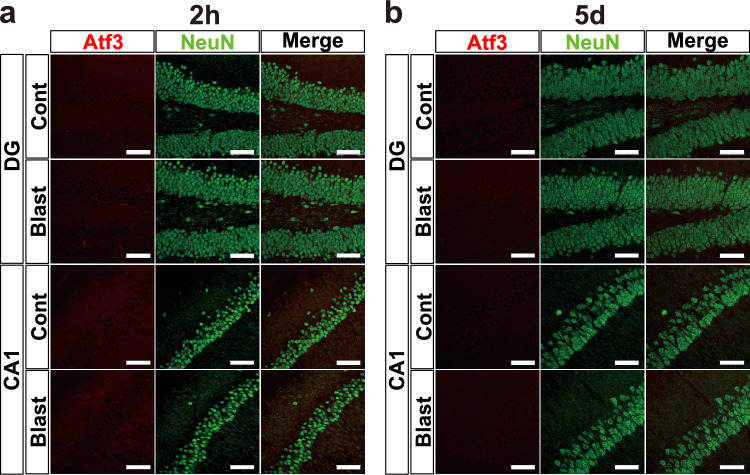
ATF3 immunoreactivity in the hippocampus. **a** At 2 h after blast exposure, ATF3 immunoreactivity was nearly absent in the dentate gyrus (DG) and CA1 regions of the hippocampus in both the control and bTBI groups. **b** At 5 days after blast exposure, similar to at 2 h post-exposure, ATF3 immunoreactivity was nearly absent in the hippocampus in both the control and bTBI groups. NeuN: neuronal nuclei. Scale bars: 50 μm

To confirm the findings of mRNA upregulation of *Atf3*, we examined the expression of Atf3 at the protein level with immunohistochemical analysis using antibodies for Atf3 and NeuN as a neuronal marker. Previous studies showed that ATF3 induction mainly occurred in neurons [35–37], but might be also induced in other cell types including immune cells [8, 38–40]. At 2 h post-exposure, ATF3 immunoreactivities in the cerebral parietal cortex (Fig. 4a) were almost absent in both bTBI and control groups, similar to the findings of mRNA. In contrast, ATF3 immunoreactivity was increased in the same region of bTBI mice after 5 days post-exposure, whereas that of the control mice was nearly absent (Fig. 4a). These results were confirmed by a two-way ANOVA with Holm-Šidák multiple-comparison test (Fig. 4b, n = 6 for each), indicating the significant main effect of blast exposure (control vs. bTBI,* F* (1, 20) = 44.43, *p* < 0.0001), significant main effect of time (2h vs. 5 d,* F* (1, 20) = 31.51, *p* < 0.0001), and significant interaction effect (*F* (1, 20) = 29.57, *p* < 0.0001). ATF3-positive cells were colocalized with NeuN, indicating that ATF3 was mainly expressed in neurons, although the possibility that ATF3 is expressed in other types of cells cannot be completely excluded. We observed little ATF3 immunoreactivity in the hippocampus in both bTBI and control groups at 2 h (Fig. 5a) and 5 days post-exposure (Fig. 5b).

### Activation of astrocytes and microglia after blast exposure

**Fig. 6:**
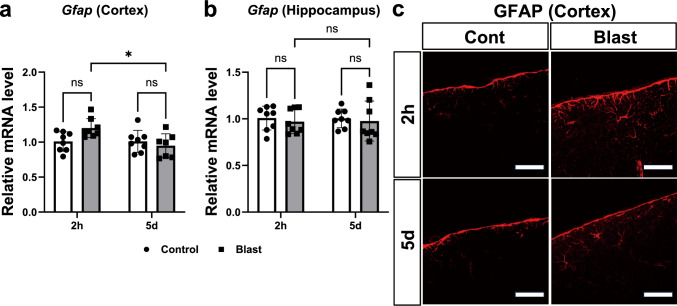
Activation of astrocytes after blast exposure. **a** In the cortex, *Gfap* mRNA levels were not significantly upregulated compared to the controls at either 2 h and 5 days after blast exposure (two-way ANOVA with Holm-Šidák multiple-comparison test, control (2h): n = 8, bTBI (2h): n = 8, control (5d): n = 8, bTBI (5d): n = 7). **b** In the hippocampus, *Gfap* mRNA levels in were indistinguishable between the groups (two-way ANOVA with Holm-Šidák multiple-comparison test, n = 8 for each group). Data are presented as mean ± standard error of the mean. **c** At 2 h after blast exposure, expression levels of GFAP protein were significantly upregulated in the cortex compared to the controls. At 5 days after blast exposure, expression of GFAP protein was significantly upregulated in the cortex compared to the controls. Scale bars: 50 μm

Neuroinflammation is partially characterized by the activation of microglia and astrocytes. An abnormal increase in astrocyte reactivity in response to injury (astrogliosis) involves increased expression of the intermediate filament proteins, GFAP with extended processes and hypertrophy [41–45]. Therefore, the mRNA levels of *Gfap* were analyzed. In the cortex (Fig. 6a), a two-way ANOVA (control (2h): n = 8, bTBI (2h): n = 8, control (5d): n = 8, bTBI (5d): n = 7) indicated that the significant main effect of time (2h vs. 5 d,* F* (1, 27) = 5.409, *p* = 0.0278), and significant interaction effect (*F* (1, 27) = 5.539, *p* = 0.0261), while no significant main effect of blast exposure (control vs. bTBI, *F* (1, 27) = 1.475, *p* = 0.2351). However, Holm-Šidák multiple-comparison test revealed no significant difference in the mRNA levels of Gfap between the blast exposure and controls at either 2 h and 5 days after blast exposure. In the hippocampus, there were no main effects (for either blast exposure or time) and no significant interaction effect (Fig. 6b; all *p* > 0.05, two-way ANOVA, n = 8 for each).

Expression of GFAP protein was significantly upregulated in the bTBI group at 2 h post-exposure in the cerebral cortex compared to the controls (Fig. 6c). At 5 days post-exposure, the increased expression level of GFAP protein in the cerebral cortex was sustained in mice with blast exposure (Fig. 6c). These results are in consistence with the results of western blot analysis (Fig. S1).

**Fig. 7:**
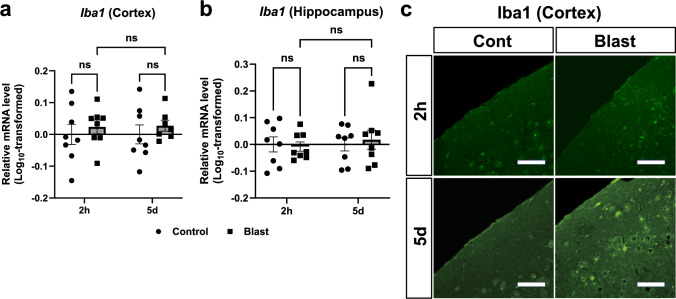
Activation of microglia after blast exposure. **a** In the cortex, *Iba1* mRNA levels were indistinguishable between the groups at either 2 h and 5 days after blast exposure (Games-Howell multiple-comparison test, control (2h): n = 8, bTBI (2h): n = 8, control (5d): n = 8, bTBI (5d): n = 7). **b** Similarly in the hippocampus, *Iba1* mRNA levels were indistinguishable between the groups (Games-Howell multiple-comparison test, n = 8 for each group). Data are presented as mean ± standard error of the mean. **c** At 2 h after blast exposure, expression levels of Iba1 protein in the cortex were indistinguishable between the groups. To the contrary, expression levels of Iba1 protein were upregulated at 5 days after blast exposure compared to the controls. Scale bars: 50 μm

Previous studies reported that microglia has critical functions in neuroinflammation after other non-blast TBIs, and is chronically activated with increased levels of Iba1 [41, 46]. Therefore, the activation of Iba1 was assessed as a marker for active microglia. A Games-Howell test (Fig. 7a; control (2h): n = 8, bTBI (2h): n = 8, control (5d): n = 8, bTBI (5d): n = 7) indicated that mRNA levels of *Iba1* were indistinguishable between the groups in both the cerebral cortex (Fig. 7a; all *p* > 0.05, n = 8 for each) and hippocampus (Fig. 7b; all *p* > 0.05, n = 8 for each).

At 2 h post-exposure, expression of Iba1 protein in cerebral cortex was indistinguishable between the bTBI and control groups (Fig. 7c). However, at 5 days post-exposure, significantly increased expression of Iba1 was observed in the cerebral cortex of bTBI mice compared to the controls (Fig. 7c). These results were in consistence with the results of western blot analysis (Fig. S1). Overall, mRNA levels of *Iba1* were not upregulated in the acute and subacute phases after blast exposure whereas the protein levels of Iba1 were upregulated in the subacute phase, but not in the acute phase.

## Discussion

The present study observed ATF3 upregulation and the activation of microglia and astrocytes in an animal model of bTBI. Therefore, bTBI-associated neuroinflammation involves transcriptional modulation of genes encoding for immune-modulatory proteins. Furthermore, our results indicated that the time course of ATF3 upregulation post-exposure seems to be distinct from that in a non-blast TBI model (i.e., closed-head weight-drop–based TBI model), which showed that upregulation of ATF3 occurs within 2–3 h post-exposure and decreases by 4 h later [9]. Similarly, ischemic stroke/reperfusion models showed that ATF3 upregulation is induced by 2 h post-exposure [9, 10, 47]. In contrast, our results showed that mRNA levels of *Atf3* were upregulated at 5 days post-exposure, but not immediately. Thus, induction of *Atf3* upregulation in bTBI may be delayed compared with non-blast TBI.

The present study showed that ATF3 was induced by bTBI, but the functions of ATF3 in bTBI has not been elucidated. However, evidence from another injury model of non-blast TBI suggest that ATF3 is essential to inhibit the transcription of numerous proinflammatory cytokines containing ATF/CREB binding sites, thus reducing neuroinflammation in the subacute phase of TBI [9]. Increased inflammation, greater infarct volume, and worsening brain function were observed after transient focal cerebral ischemia in the CNS of ATF3-deficient mice [12, 48]. ATF3-deficient mice showed significantly increased induction of several immune genes and abundance of immune cells compared to the induction observed in wild-type mice using the closed-head weight-drop model [9]. Furthermore, ATF3 deficiency enhanced TBI-induced paresis and hematoma formation, suggesting that ATF3 limits these injury outcomes in wild-type mice [9]. Conversely, persistent ATF3 expression induced by excessive reactive oxygen species or endoplasmic reticulum stress may have detrimental effects, overwhelming the initial protective action [49]. Therefore, the effects of ATF3 on inflammation might depend on the specific cellular environments under stress.

Notably, our immunohistochemical study indicated that ATF3 staining was predominantly cytoplasmic, with a low signal in the nucleus (Fig. 6). As a transcription factor, ATF3 expression was expected in nuclear. In regard to this point, a previous study indicated different intracellular localization of ATF3 that shifts from nuclear (in neuronal progenitors) to cytoplasmic (in more mature neurons) during neuronal differentiation [50]. Although the roles of cytoplasmic ATF3 is not understood, they have important roles in response after injury with the impact on neuronal differentiation, survival, network formation, and regeneration [50]. Further research is warranted to understand the involvement of ATF3 in the nuclear versus cytoplasmic localization.

We observed activation of brain-resident types of glia in bTBI. In the acute phase of other TBIs, quiescent glial cells in the cerebral cortex become rapidly activated via a process identified as “reactive gliosis” and cause the generation and release of inflammatory mediators such as cytokines and chemokines [51]. Therefore, astrocytes and microglia are considered to have key functions in initiating neuroinflammation. Notably, previous studies indicated that activated astrocytes and microglia in the brain can upregulate ATF3 on injury [52, 53]. The present study showed that the protein expression of GFAP in the cortex was rapidly upregulated and sustained during the subacute phase after blast exposure (Fig. 6c, S1). The protein expression of Iba1 in the cortex was also upregulated during the subacute phase after blast exposure (Fig. 7c, S1).

However, the time courses of glial mRNA transcriptions were slightly different from that of glial protein detection. Holm-Šidák multiple-comparison test indicated that *Gfap* mRNA levels in the cortex were indistinguishable between the bTBI and control groups at both 2 h and 5 days post-exposure and *Iba1* mRNA levels in the cortex were also indistinguishable between groups. Although we do not know the reason for the discrepancy between glial mRNA transcript and glial protein detection, such phenomena have sometimes been reported before in vivo and in vitro [54–59]. For example, GFAP protein expression has been preceded by *Gfap* transcript expression [56, 57], likely due to a translational mechanism [54]. In addition, changes in the translational mechanism delaying GFAP protein production also implies a distinctive function specifically for *Gfap* mRNA [60].

In conclusion, this study established the involvement of ATF3 in bTBI. So far, no research has reported the actions of ATF3 in the subacute phase of TBI. More time points are required to elucidate the exact time course, but the present results show a distinctly different time dependence of ATF3 upregulation between bTBI and non-blast TBIs. Consequent to recent medical interest in the time course of neuroinflammation in TBI, further research including more time points is warranted to understand the involvement of ATF3 in the acute versus subacute neuroinflammation. Another limitation is that only 8-week-old male mice were used in this study. Since sex and age affect neuroinflammatory responses, further research including female or aged mice would be necessary in the future.

## Supplementary Information

Below is the link to the electronic supplementary material.

Supplementary file1 (EPS 1777 KB)

Supplementary file2 (DOCX 23 KB)

## Data Availability

Data will be made available on request.
