# Supplementary material for: Induction of activating transcription factor 3 (ATF3) in the cerebral cortex of a mouse model of blast-induced traumatic brain injury
Source: Hum Cell. 2026 Apr 7;39(4):60. doi: 10.1007/s13577-026-01377-1 (PMC13056789; doi:10.1007/s13577-026-01377-1)
Supplement: Supplementary file 2 — Supplementary file2 (DOCX 23 KB) [file 13577_2026_1377_MOESM2_ESM.docx]

**Materials and Methods**

**Western blot analysis**

Western blot analysis of protein lysates from the cortex was performed as previously described [1]. Briefly, mice were euthanized by cervical dislocation and tissues were dissected. Proteins extracted were subjected to SDS-PAGE, and then proteins were transferred onto polyvinylidene fluoride membranes (Immobilon-P, Millipore, Bedford, MA). The blots were immunoreacted with primary antibodies including anti-ATF3 (ab207434; rabbit monoclonal, Abcam, Cambridge, UK), anti-GFAP (#12389, rabbit monoclonal, Cell Signaling Technology, Danvers, Ma) and anti-Iba1 (ab178847; rabbit monoclonal, Abcam), and anti-β-actin (A5441, mouse monoclonal, Sigma, St. Louis, MO) antibodies. The secondary antibodies used were horseradish peroxidase (HRP)-linked anti-rabbit IgG (#7074, goat polyclonal, Cell Signaling Technology) and horseradish peroxidase (HRP)- linked anti-mouse IgG (#7076, horse polyclonal, Cell Signaling Technology). The immunostained protein bands were visualized with a chemiluminescent detection system, Immunostar (Wako, Osaka, Japan). Blot images were captured by a luminescent image analyzer (Amersham Imager 600, GE Healthcare, Tokyo, Japan). Samples from four mice per experimental condition were examined in each experiment.

**Statistical analysis**

Statistical analyses were performed using GraphPad Prism 9.5.1 (GraphPad Software, La Jolla, CA). We examined 4 mice per condition (total 16 mice). The levels of expression were normalized to those of the corresponding β-actin as a reference protein. One sample t-test was used to determine whether the mean of differences for each group was different from the control (2h, control). The results are presented as the mean ± standard error of the mean. The significance level was set at less than 0.05, indicating that *p*-values below this threshold were considered statistically significant.

**Results**

We examined the Atf3 protein expression levels in the cortex by western blot analysis (Fig. S1). At 2 hours post-exposure, the levels of Atf3 expression was indistinguishable between bTBI and control groups in the cerebral cortex (Fig. S1; *p* > 0.05, one sample *t*-test, n = 4 for each). In contrast, Atf3 expression level was significantly increased in bTBI mice after 5 days post-exposure compared to that of the control mice (Fig. S1; *t* = 5.591, *p* = 0.0113, one sample *t*-test, n = 4 for each). Next, we examined the levels of GFAP expression (Fig. S1). At 2 hours post-exposure, GFAP expression level was significantly increased in bTBI mice compared to that of the control mice (Fig. S1; *t* = 3.204, *p* = 0.0492, one sample *t*-test, n = 4 for each). Similarly, the levels of GFAP expression was significantly increased in bTBI mice after 5 days post-exposure compared to that of the control mice (Fig. S1; *t* = 4.616, *p* = 0.0191, one sample *t*-test, n = 4 for each). These results are in consistence with the results of immunohistochemical analysis (Fig. 6C). We then examined the levels of Iba1 expression (Fig. S1). At 2 hours post-exposure, the levels of Iba1 expression was indistinguishable between bTBI and control groups in the cerebral cortex (Fig. S1; *p* > 0.05, one sample *t*-test, n = 4 for each). In contrast, Iba1expression level was significantly increased in bTBI mice after 5 days post-exposure compared to that of the control mice (Fig. S1; *t* = 3.301, *p* = 0.0457, one sample *t*-test, n = 4 for each). These results are in consistence with the results of immunohistochemical analysis (Fig. 7c). The expression level of β-actin was unaltered after injury (Fig. S1; all *p* > 0.05, one sample *t*-test, n = 4 for each).

**References**

[1] Satoh Y, Endo S, Nakata T, Kobayashi Y, Yamada K, Ikeda T, Takeuchi A, Hiramoto T, Watanabe Y, Kazama T. ERK2 contributes to the control of social behaviors in mice. J Neurosci, 2011; 31: 11953-67.

**Figure legend**

FIG. S1. Western blot analysis for Atf3, Gfap, Iba1, and β-actin

1. Representative western blots showing expression of Atf3, GFAP, and Iba1 protein expression of control (2h), blast (2h), control (5d), and blast (5d) mice (n = 4 for each). β-actin was the loading control. (b) To evaluate expression levels, protein band intensities were normalized to those of the loading control (β-actin). Data were analyzed by one-sample *t*-test: **p* < 0.05 (n = 4 for each).
